# Supplementary material for: HIVprotI: an integrated web based platform for prediction and design of HIV proteins inhibitors
Source: J Cheminform. 2018 Mar 9;10:12. doi: 10.1186/s13321-018-0266-y (PMC5845081; doi:10.1186/s13321-018-0266-y)
Supplement: Supplementary file 2 — Additional file 2. Source code of HIVProtI web server. [file 13321_2018_266_MOESM2_ESM.zip › HIVprotI_Source-code/hivprotI/weka-3-6-6/doc/weka/classifiers/lazy/kstar/KStarWrapper.html]

KStarWrapper


---


|  |  |  |
| --- | --- | --- |
| **Overview** \| **Package** \| **Class** \| **Tree** \| **Deprecated** \| **Index** \| **Help** \| **Weka's home** | |  |
| **PREV CLASS**   NEXT CLASS | **FRAMES**    **NO FRAMES**     **All Classes** |  |
| SUMMARY: NESTED \| FIELD \| CONSTR \| METHOD | DETAIL: FIELD \| CONSTR \| METHOD |  |


---


## weka.classifiers.lazy.kstar Class KStarWrapper

```
java.lang.Object
  weka.classifiers.lazy.kstar.KStarWrapper
```

**All Implemented Interfaces:**: RevisionHandler

---

``` public class KStarWrapper extends java.lang.Object implements RevisionHandler ```

---

| **Field Summary** | |
| --- | --- |
| `double` | `actEntropy`             used/reused to hold the actual entropy |
| `double` | `avgProb`             used/reused to hold the average transformation probability |
| `double` | `minProb`             used/reused to hold the smallest transformation probability |
| `double` | `randEntropy`             used/reused to hold the random entropy |
| `double` | `sphere`             used/reused to hold the sphere size |


**Constructor Summary**

`KStarWrapper()`


| **Method Summary** | |
| --- | --- |
| `java.lang.String` | `getRevision()`             Returns the revision string. |

**Methods inherited from class java.lang.Object**

`equals, getClass, hashCode, notify, notifyAll, toString, wait, wait, wait`

**Field Detail**

### sphere

```
public double sphere
```

:   used/reused to hold the sphere size

---


### actEntropy

```
public double actEntropy
```

:   used/reused to hold the actual entropy

---


### randEntropy

```
public double randEntropy
```

:   used/reused to hold the random entropy

---


### avgProb

```
public double avgProb
```

:   used/reused to hold the average transformation probability

---


### minProb

```
public double minProb
```

:   used/reused to hold the smallest transformation probability


**Constructor Detail**

### KStarWrapper

```
public KStarWrapper()
```


**Method Detail**

### getRevision

```
public java.lang.String getRevision()
```

:   Returns the revision string.

    :   **Specified by:**: `getRevision` in interface `RevisionHandler`
    :   **Returns:**: the revision


---


|  |  |  |
| --- | --- | --- |
| **Overview** \| **Package** \| **Class** \| **Tree** \| **Deprecated** \| **Index** \| **Help** \| **Weka's home** | |  |
| **PREV CLASS**   NEXT CLASS | **FRAMES**    **NO FRAMES**     **All Classes** |  |
| SUMMARY: NESTED \| FIELD \| CONSTR \| METHOD | DETAIL: FIELD \| CONSTR \| METHOD |  |


---
